# Eight-year Review of a Clubfoot Treatment Program in Pakistan With Assessment of Outcomes Using the Ponseti Technique: A Retrospective Study of 988 Patients (1,458 Clubfeet) Aged 0 to 5 Years at Enrollment

**DOI:** 10.5435/JAAOSGlobal-D-22-00022

**Published:** 2022-04-25

**Authors:** Sadia Ahmed, Shazia Moosa, Ammar Ali Muhammad, Sundus Iftikhar, Mansoor Ali Khan, Muhammad Amin Chinoy, Lubna Samad

**Affiliations:** From the Indus Hospital and Health Network, Karachi, Pakistan (Ahmed and Moosa); Interactive Research and Development, Karachi, Pakistan (Muhammad, Iftikhar, and Samad); the Department of Orthopaedics (Khan and Chinoy), and the Department of Paediatric Surgery, The Indus Hospital, Karachi, Pakistan (Samad).

## Abstract

To conduct an 8-year retrospective review of a clubfoot treatment program using the Ponseti technique with close monitoring of outcomes.

**Methods:**

Between October 2011 and August 2019, 988 children with 1,458 idiopathic clubfeet were enrolled, ages ranging from new born up to 5 years. Ponseti treatment was used, and progress was monitored by comparing mean Pirani scores at enrollment (P1), initiation of bracing (P2), and end of treatment (P3) or most recent visit (P4) for children under treatment.

**Results:**

A statistically significant reduction in Pirani scores was noted (*P* < 0.001) for all feet. For 320 feet completing treatment (213 children), the mean Pirani scores reduced from P1: 3.8 (±1.1) to P2: 1.1 (±0.6) and finally to P3: 0.6 (±0.3). Four hundred sixteen children are currently undergoing bracing. Higher education of the head of household and male sex of the child were markedly associated with improved outcomes in foot correction status. Correction was obtained with a mean of 5.8 casts per foot, the tenotomy rate was 68.2%, and the mean duration of bracing in children completing treatment was 3.6 years (±0.9). No surgical correction, other than tenotomy, was required. Relapse was noted in 12.1% of the total enrolled feet, and 32.0% children were lost to follow-up from the entire cohort of 988 children.

**Conclusion:**

Clubfoot treatment requires long-term follow-up. A dedicated clubfoot program is effective in maintaining continuity of care by encouraging adherence to treatment.

Global incidence of clubfoot is 1.24 per 1,000 live births, demonstrating prevalence in all six inhabited continents of the world.^1,2,3,4,5,6^ It is the commonest musculoskeletal deformity and the seventh most common congenital birth defect.^7^ Clubfoot may be associated with neuromuscular disease, syndromes, and chromosomal or congenital abnormalities; however, isolated clubfoot deformity in an otherwise normal child is labeled idiopathic and has four components—equinus, varus, adductus, and cavus.

Treatment of idiopathic clubfoot using the Ponseti method is considered “gold standard” across the world.^2,8-10^ Dr Ignacio Ponseti, an orthopaedic surgeon, pioneered this nonsurgical method as a simple, inexpensive, easily adopted, and applied outpatient technique for correction of clubfoot.^11^ Popularity of this method is steadily increasing, and its judicious use has yielded excellent results, with surgical correction rarely required.^12^ Orthopaedic societies in more than 45 countries across the globe have endorsed Ponseti treatment,^8^ and 113 of the 193 United Nations member countries report some evidence of Ponseti activity.^13^ It is important to note that low- and middle-income countries (LMICs) are host to more than 90% of clubfoot cases. A study in 2015 documented that only 15% of clubfoot cases accessed Ponseti treatment^14^; therefore, a majority of untreated or undertreated cases lead to neglected clubfoot and consequent life-long disabilities contributing to the health burden in developing countries. In Pakistan, approximately 7,500 children are born with clubfoot every year; this approximation is based on calculations using World Bank figures for population and a crude birth rate.^14^ The poorly structured health delivery system translates into a high percentage of these children being left untreated; this has notable social and economic consequences in a predominantly agricultural, developing country where physical labor is the main form of employment for the majority.

Stand-alone clubfoot programs managed by nonsurgeon clinical officers have been implemented; however, lack of supplies and reliable referral service for surgery in complicated cases were identified as main issues.^15,16^ Better outcomes were observed when clubfoot was treated in dedicated Ponseti clinics embedded within orthopaedic services, with lower recurrence rates and prompt on-site referrals when required^17^; this is the model that we adopted for our clubfoot program, headquartered in the country's largest city. Healthcare access is a major barrier in low-resource settings^15,18^; thus, it is vital to use the opportunity and provide the required comprehensive care when children with abnormalities present to a tertiary care facility. The College of Physicians and Surgeons Pakistan recognizes 56 orthopaedic residency training programs based at public and private tertiary hospitals across the country^19^; this provides the opportunity to establish clubfoot clinics within facility-based orthopaedic services with the added advantage of training upcoming orthopaedic surgeons in this method. Establishment of integrated programs contributes to strengthening of health systems as compared with vertical programs that tend to work in silos.^15,16^

This study aims to determine the effectiveness of the Ponseti technique in children with idiopathic clubfoot enrolled in the program by comparing the mean Pirani scores of feet over the course of treatment. It also intends to examine the association of factors with foot correction status along with presenting the clinical outcomes of those enrolled children who have completed the entire course of treatment. In addition, the integral program processes for the purpose of monitoring and evaluation have been reviewed and presented.

## Methods

### Program Description

The “Pehla Qadam-PQ (First Step) Program” was initiated in August 2011 with a preliminary preparation phase followed by commencement of a clubfoot treatment clinic in October 2011, at The Indus Hospital (TIH), Karachi, the flagship campus of the Indus Hospital & Health Network (IHHN). When the program was in its nascent phase, only children up to the age of 1 year were enrolled for treatment. With passage of time, our referrals increased by word of mouth and older children also presented to our clinic with untreated clubfoot. To save them from future disability, we gradually increased our age limit for inclusion to 5 years.

IHHN provides all treatment free of cost; for children in the PQ program, braces are also provided and a transport allowance is offered to families to encourage adherence during the long course of clubfoot treatment. Overall management of clubfoot patients using the Ponseti method includes case selection after accurate diagnosis, sequential cast applications, Achilles tenotomy (if required), maintenance bracing, periodic assessment to prevent relapse, managing associated complications, and monitoring of outcomes. Pirani scoring is used to determine the severity of clubfoot and to aid in clinical decision making.^20^ Ultimate objective of the treatment is to help the child attain good clinical outcomes allowing him to lead a normal life. After treatment completion, patients are followed up annually for 5 years.

#### Personnel and Training

The program team includes a coordinator responsible for developing program tools, training health workers, analyzing data, and managing the program. Health workers are responsible for counseling families, obtaining informed consent from caregivers, enrolling children, maintaining photographic record, data collection, and entry. The clinical team comprises orthopaedic surgeons and residents from the Department of Orthopaedics at TIH who have been trained in the Ponseti method; they screen children at the initial visit, conduct Pirani scoring of their feet at each subsequent visit, apply casts, conduct tenotomy, and assign braces; a plaster technician assists the doctors during plaster application.

#### Program Tools

Brochures include information about clubfoot and the PQ program, plaster care and removal, and handling of braces. Standardized proformas are used to document patient details and outcomes.

#### Clinic Routine

PQ outpatient clinic is conducted twice a week where new patients are registered and returning patients are assessed for progress.

#### Data Documentation

Demographic data are collected from each patient at the time of enrollment. At each clinic visit, photographs are obtained and Pirani scores recorded, which range from 0 to 6 in half-point intervals, where 0 is a normal foot and 6 is the most severe deformity.^5,15,21^ Data are entered on the hospital database and in the International Clubfoot Registry. Monthly reports are generated and analyzed.

#### Program Processes

##### Casting

Management consists of an initial “treatment phase” involving gentle manipulation of the child's foot at each visit to stretch the ligaments and tendons followed by plaster cast application in the new stretched position; this routine is carried out weekly for 6 to 8 weeks. Plaster of Paris casts are used in children younger than 3 years at the time of presentation, whereas synthetic casts are applied in older children for whom the Ponseti technique has been modified by increasing the number of casts required to achieve the desired correction (Pirani score 0 to 1) and by keeping each cast on for 2 weeks.^20,22^ Families are counseled to remove the Plaster of Paris cast 2 hours before the next appointment.^23^ This allows clinic time to be used more efficiently. Synthetic casts are removed in the clinic by the plaster technician.

##### Tenotomy

Based on the surgeon's assessment, percutaneous tenotomy to release the Achilles tendon may be conducted as an outpatient procedure under local anesthesia. After tenotomy, the affected foot is placed in a cast for 3 weeks in the fully corrected position before initiating bracing.

##### Bracing

In the “maintenance phase,” children are provided with braces, worn 23 hours a day for 2 to 3 months, followed by night-time bracing until the age of 4 years. Children are followed up in the clinic after 1 month of initiation of braces and then every 3 months for 4 years. More frequent visits may be scheduled for severe or poorly compliant cases. The decision to discontinue bracing is based on clinical judgment; in some cases, the duration may be extended by a few months.^21,24^ Children older than 1 year at enrollment are advised bracing for a duration of at least 2 years.^8,16^ Daily exercises are taught to the parents to help reduce foot rigidity.

##### Relapse

Appearance of cavus, adductus, varus, or equinus, depicted by worsening Pirani scores, is considered a relapse.^4,25^ In such cases, manipulation and casting are reinitiated followed by tenotomy, if needed.^20^

##### Loss to Follow-up

Families are counseled regularly to encourage treatment adherence. If a patient misses an appointment, the health worker calls the family on the same day to reschedule the appointment. Failure to visit the clinic after three consecutive rescheduled appointments is labeled “lost to follow-up,” which also includes families refusing to continue treatment at any stage. Reasons for refusal are documented, and if geographical distance is the determining barrier, efforts are made to direct the family to a treatment facility closer to their residence. Noncontactable families are also considered “lost to follow-up.”

##### Completion of Treatment

All children undergoing the specified duration of treatment resulting in notable improvement in clinical outcomes are considered to have completed their treatment. These specific clinical parameters include cosmetic appearance, flexibility, pain, position of the foot, squatting, walking without a limp, running, wearing normal shoes, and carrying out daily activities with ease.

### Study Description

The duration of this retrospective study is from October 2011 to August 2019.

#### Patient Selection

##### Inclusion Criteria

(1) All children completing Ponseti treatment.
(2) Children completing casting and progressing to braces (under treatment).

##### Exclusion Criteria

**Figure 1:**
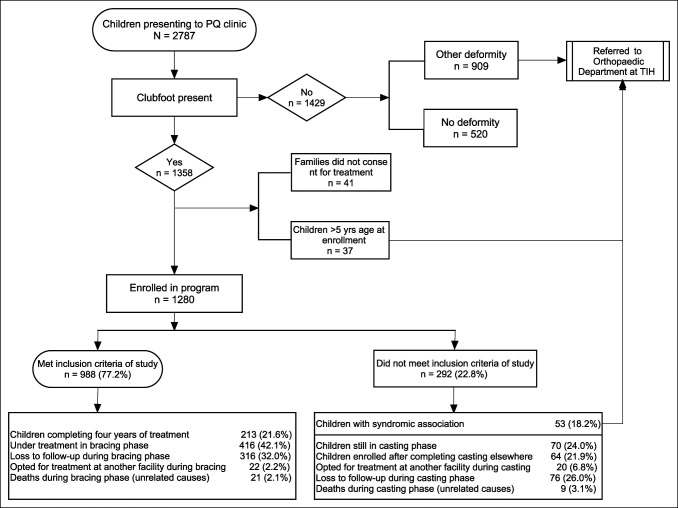
Flowchart showing a clubfoot treatment program in Pakistan, 2011 to 2019. PQ = Pehla Qadam, TIH = the Indus Hospital.

Children older than 5 years at enrollment, those with syndromic associations, those still in the casting phase at the study cutoff date, or those enrolled in our program during the bracing phase of treatment are excluded from this study (Figure 1).

#### Ethical Approval

Approval was granted by the Institutional Review Board of TIH.

#### Informed Consent

Informed consent was obtained from parents of all children included in this study.

#### Outcome Assessment

For the purpose of this study, Pirani scores at three points during treatment were noted to assess the efficacy of the Ponseti technique—(P1) at enrollment, (P2) at initiation of bracing, and (P3) at the end of treatment or (P4) at the most recent clinic visit for children under treatment. Patients completing full treatment were assessed to see if their duration of treatment was within the acceptable range described by Ponseti, that is, “timely correction” or “delayed correction,” and factors associated with foot correction status were examined. Posttreatment clinical outcomes were also recorded.

### Data Analysis

Data were analyzed using Stata version 14. Normality was assessed for all the quantitative variables. Mean (SD) and median (interquartile range) were reported for quantitative variables as appropriate. All the categorical variables were presented using frequencies and percentages. Age categories were studied with mean cast numbers using one-way analysis of variance. Repeated measures analysis of variance was used to assess the effectiveness of the Ponseti method by comparing the means of Pirani scores at the three specified time points. Foot correction status of children with completed treatment was categorized as “timely correction” and “delayed correction.” The chi square test was used to study the association of different factors with correction status. All tests were two-sided, and *P* values <0.05 were considered significant.

## Results

During the study period, 2,787 children were brought to the PQ clinic with suspected clubfoot. On initial screening (Figure 1), 909 were diagnosed with conditions other than clubfoot (cerebral palsy, vertical talus, myelomeningocele, developmental dysplasia of the hip, metatarsus adductus, spina bifida, genu varus, genu valgus, and others), while 520 were assessed to be normal; 41 children came from remote locations from where frequent commute was not possible and 37 were older than 5 years. Thus 1,280 children were consented and enrolled in the program. The eligibility criteria for this study were not met by 292 children; therefore, 988 children with 1,458 affected feet were included. Treatment completion was achieved in 213 children (21.6%); 416 children (42.1%) are currently in the bracing phase, 316 children (32.0%) were lost to follow-up, 22 children (2.2%) transferred care to another facility, and 21 children (2.1%) died while in the bracing phase because of unrelated causes.

### Demographic Data

Most of the children were male (n = 760, 76.9%) and the age at enrollment ranged from 1 day to 54 months (median = 3.5 months, interquartile range = 1.48 to 9.88 months); 44.5% of the enrolled children belonged to the 0 to 3 months age category (Table 1). Approximately half of the enrolled children had unilateral foot involvement (n = 518) with the right foot affected in 295 cases and left in 223 cases. A family history of clubfoot was elicited for 222 children, with first-degree and second-degree or third-degree relatives affected in 32.0% and 68.0%, respectively.

**Table 1:** Demographics of Clubfoot Children Treated With the Ponseti Technique in a Clubfoot Treatment Program in Pakistan, 2011 to 2019

Variables	n (%)
Total children (N = 988)	
Children with completed treatment	213 (21.6)
Children in the bracing phase	775 (78.4)
Sex	
Male	760 (76.9)
Female	228 (23.1)
Affected feet (total = 1,458)	
Bilateral	470 (47.6)
Unilateral	518 (52.4)
Right	295
Left	223
Age at enrollment	
0 to 3 mo	440 (44.5)
>3 mo to 1 yr	345 (34.9)
>1 yr to 5 yrs	203 (20.5)
Ethnicity	
Muhajir	380 (38.5)
Sindhi	140 (14.2)
Punjabi	121 (12.2)
Pathan	97 (9.8)
Balochi	16 (1.6)
Others	234 (23.7)
Family history of clubfoot	
First-degree relatives	71 (7.2)
Second-degree and third-degree relatives	151 (15.3)
None	766 (77.5)
Education status of the head of household	
Not educated	309 (31.3)
3 to 10 yrs of education	458 (46.4)
>10 yrs of education	186 (18.8)
Informal education	30 (3.0)
Not known	5 (0.5)
Occupation of father	
Skilled laborer	350 (35.4)
Laborer	284 (28.7)
Factory worker	223 (22.6)
Agriculture/fishery work	50 (5.1)
Unemployed	7 (0.7)
Others	74 (7.5)
Source of referral	
Word of mouth	686 (70.0)
Other hospitals	167 (16.9)
Indus Hospital: other departments	84 (7.9)
Advertisement/social media	29 (2.9)
Lady health worker/maternity home/vaccinator	22 (2.2)

### Pirani Scores

**Figure 2:**
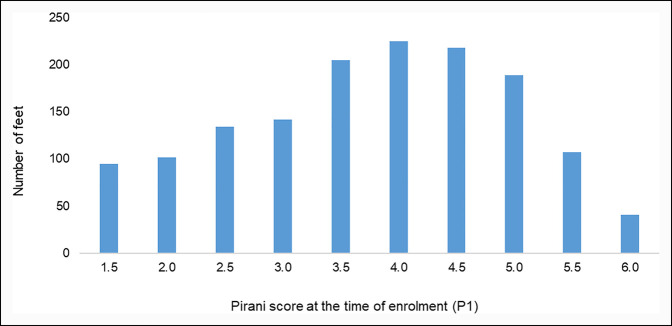
Graph showing the Pirani score of clubfeet at the time of enrollment in a clubfoot program in Pakistan, 2011 to 2019.

Of the 1,458 feet, more than half presented with a Pirani score (P1) between 3.5 and 5.0 (Figure 2). Mean Pirani scores for feet completing treatment and those progressing to braces are summarized in Table 2. Among the feet completing treatment, a significant reduction (*P* < 0.001) in mean Pirani scores was noted from P1: 3.8 (±1.1), to P2: 1.1 (±0.6) and finally to P3: 0.6 (±0.3). Furthermore, a statistically significant reduction in mean Pirani scores was also noted for feet still under treatment.

**Table 2:** Pirani Scores (PS) in Enrolled Feet During the Course of Ponseti Treatment in a Clubfoot Program in Pakistan, 2011 to 2019

Category 1: Feet Completing Treatment				
No. of Feet	P1	P2	P3	*P* Value^a^
Total feet (n = 320)	3.8 ± 1.1	1.1 ± 0.6	0.6 ± 0.3	<0.001
Right foot (n = 174)	3.8 ± 1.1	1.1 ± 0.6	0.5 ± 0.3	<0.001
Left foot (n = 146)	3.9 ± 1.2	1.2 ± 0.6	0.6 ± 0.3	<0.001

Category 2: Feet Under Treatment (Progressing to Braces)				
No. of Feet	P1	P2	P4	*P* Value^a^
Total feet (n = 1,138)	3.7 ± 1.2	1.1 ± 0.5	0.8 ± 0.5	<0.001
Right foot (n = 591)	3.7 ± 1.2	1.0 ± 0.5	0.8 ± 0.5	<0.001
Left foot (n = 547)	3.8 ± 1.2	1.1 ± 0.5	0.8 ± 0.6	<0.001

P1 = mean PS at the start of treatment, P2 = mean PS at the start of bracing, P3 = mean PS at the end of treatment, P4 = mean PS at the most recent clinic visit

Data are presented as mean ± SD.

a Repeated measures analysis of variance.

### Casting, Tenotomies, and Bracing

Correction was obtained with a mean of 5.8 casts per foot (±3.8), with most (89.7%) corrected with 10 casts or less. Of the 150 feet that required more than 10 casts, 87 were rigid, whereas treatment was interrupted in 63 feet leading to higher cast numbers. A significantly higher mean number of casts (*P* < 0.001) were seen in children older than 1 year (7.2, ±5.0) as compared with those of age 0 to 3 months (5.3, ±3.2) and >3 months to 1 year (5.6, ±3.5). Tenotomies were conducted in 995 feet (68.2%), with a single procedure done in 939 feet and repeat tenotomies (up to 3) required in 56 feet, with no reported complications. No surgical correction, other than tenotomy, was required in our cohort. The mean duration of bracing for 213 children who completed treatment was 3.6 years (±0.9). Table 3 summarizes the mean treatment duration of different age categories in the treated cohort, and it can be noted that the mean duration of treatment is comparatively less (3.3, ±1.0) for older children. No association was seen between the number of casts and the brace duration (*P* = 0.31).

**Table 3:** Mean Brace and Treatment Duration of Different Age Categories in the Treated Cohort in a Clubfoot Program in Pakistan, 2011 to 2019

Age Categories	No. of Children, n (%)	Brace Duration (yrs), Mean (SD)	Treatment Duration (yrs), Mean (SD)
0 to 3 mo	94 (44.1)	3.9 (0.7)	4.6 (0.9)
>3 mo to 1 yr	71 (33.3)	3.8 (0.7)	4.4 (0.8)
>1 yr to 5 yrs	48 (22.5)	2.8 (1.0)	3.3 (1.0)
Total	213 (100)	3.6 (0.9)	4.2 (1.0)

### Relapse

Relapse during the bracing phase was noted in 177 feet (12.1%), and in these cases, casting was reinitiated. Of these, multiple relapses were seen in 27 feet (twice in 26 feet and thrice in one foot).

### Loss to Follow-up

**Figure 3:**
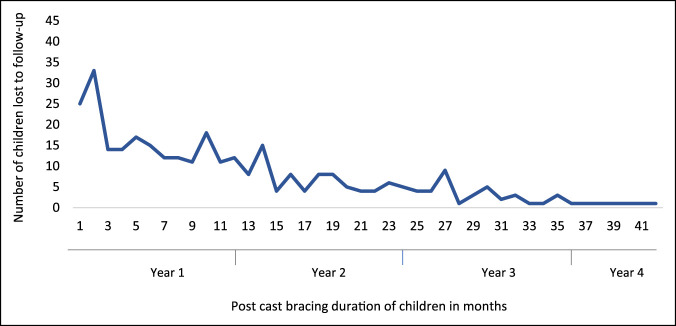
Graph showing loss to follow-up during the bracing phase in a clubfoot program in Pakistan, 2011 to 2019.

Initially, 528 children (53.4%) were assessed to fall in the category of “lost to follow-up,” of which 212 children were retrieved through active contact and counseling by PQ health workers, whereas final loss to follow-up was documented in 32.0% children (n = 316). Of the 316 children lost to follow-up, most of them (63.9%) occurred during the first year of bracing followed by 23.7%, 10.1%, and 2.2% in the second, third, and fourth or fifth years, respectively (Figure 3). The commonest reason given was relocation of family to another city (n = 111, 35.1%), followed by refusal to continue treatment (n = 51, 16.1%) and domestic issues (n = 8, 2.5%). The remaining (n = 146, 46.2%) were not contactable, so the reason remains unknown. A mortality rate of 2.1% (n = 21) was documented, with reasons reported by parents in 20 children: respiratory causes (n = 7), febrile convulsions (n = 6), acute diarrhea (n = 4), and drowning, head injury, and hydrocephalus (1 each).

### Factors Affecting Correction Status in Children Completing Ponseti Treatment

Of 213 children completing treatment, 177 (83.1%) achieved timely correction, whereas correction was delayed in 36 children. Reasons for delayed correction include interruption in treatment in 31 children, poor compliance in 3 children, and 2 children had rigid feet. “Education of the head of household” and “sex of the child” were found to be significantly associated with the foot correction status (Table 4).

**Table 4:** Association of Foot Correction Status With Independent Variables in Children Completing Ponseti Treatment (n = 213) in a Clubfoot Treatment Program in Pakistan, 2011 to 2019

Independent Variable	Timely Correction, n (%)	Delayed Correction, n (%)	*P* Value^a^
Sex			
Male	137 (86.7)	21 (13.3)	0.02^b^
Female	40 (72.7)	15 (27.3)	
Age of children at enrollment			
0 to 3 mo	74 (78.7)	20 (21.3)	0.31
>3 mo to 1 yr	61 (85.9)	10 (14.1)	
>1 yr to 5 yrs	42 (87.5)	6 (12.5)	
Ethnicity			
Muhajir	84 (86.6)	13 (13.4)	0.54
Sindhi	22 (73.3)	8 (26.7)	
Punjabi	17 (80.9)	4 (19.1)	
Pathan	21 (87.5)	3 (12.5)	
Balochi	2 (100.0)	0 (0.0)	
Others	31 (79.5)	8 (20.5)	
Family history of clubfoot			
First-degree relatives	10 (71.4)	4 (28.6)	0.46
Second-degree and third-degree relatives	13 (86.7)	2 (13.3)	
None	154 (83.7)	30 (16.3)	
Education status of the head of household			
Uneducated	37 (72.6)	14 (27.4)	0.05^b^
3 to 10 yrs of education	92 (83.6)	18 (16.4)	
>10 yrs of education	38 (90.5)	4 (9.5)	
Informal education	10 (100.0)	0 (0.0)	
Occupation of father			
Skilled laborer	98 (83.8)	19 (16.2)	0.79
Laborer	27 (87.1)	4 (12.9)	
Factory worker	33 (82.5)	7 (17.5)	
Agriculture/fishery work	6 (66.7)	3 (33.3)	
Unemployed	1 (100.0)	0 (0.0)	
Others	12 (80.0)	3 (20.0)	
Source of referral			
Word of mouth	126 (85.7)	21 (14.3)	0.47
Other hospital	26 (78.8)	7 (21.2)	
Indus Hospital	11 (68.8)	5 (31.2)	
Advertisement/social media	10 (83.3)	2 (16.7)	
Lady health worker/maternity home/vaccinator	4 (80.0)	1 (20.0)	
Affected foot			
Unilateral	85 (80.2)	21 (19.8)	0.26
Bilateral	92 (86.0)	15 (14.0)	

a Chi square test.

b *P* value ≤ 0.05.

### Clinical Outcomes in Treated Children

All children who completed the Ponseti treatment demonstrated notable improvement in all the clinical parameters mentioned earlier.

## Discussion

We present the largest, single-facility series of clubfoot patients systematically followed up in an LMIC setting where data were collected with strict compliance to program guidelines. This provides us with a unique opportunity to analyze and present findings from a resource-limited setting with rigorous standards of data compilation.

The age at initiation of casting is a key factor in the success of the treatment; although casting can be initiated as early as the age of 1 week, good results are generally also seen when started before 2 years.^2,18,26,27^ However, the Ponseti method has also been used to manage older children, even those with neglected or rigid clubfoot, with good outcomes by increasing the number and duration of casts.^6,22,28,29^ Ayana et al^20^ demonstrated successful clubfoot correction in children from the age of 2 to 10 years, reporting a correlation between age and an increasing number of casts. We report similar findings from our cohort; although initial successful correction was achieved for all affected feet with a mean of 5.8 casts per foot, a markedly higher mean number of casts were required in children older than 1 year. This decreases the need for surgical correction, as reflected by the fact that none of our treated children required any surgeries, apart from tenotomy and has also been reported by others.^20,29^ An upper age limit for using the Ponseti method remains to be determined.^5,6^

Achilles tenotomy rates range widely from 37% to 97%.^4,15,29-31^ In our study, tenotomy was required in 68.2% feet; a small number of feet required redo tenotomies, mainly to address relapse. Failure to adequately divide the tendon may explain the need for redo tenotomy. Although complications have historically been reported,^4,31,32^ none occurred in our considerably large patient population.

The real challenge remains ensuring adherence to treatment during the long duration of bracing, which allows the correction to be maintained. Clubfoot has a stubborn tendency to recur if bracing is inadequate, even after achieving perfect initial correction through casting with or without tenotomy.^11,24^ Because correction of the deformity is visible to the parents, they tend to think that their battle is over. Noncompliance to braces remains the commonest cause of relapse,^6,24,33^ with rates ranging from 13.7% to 28%.^25,34,35^ Our relapse rate of 11.7% was attributable to either poor compliance or rigid feet. Effective counseling and guidance of parents, regular contact with the family to encourage timely follow-up, uninterrupted supply of treatment materials, and encouraging interaction between parent groups were critical factors in promoting adherence to treatment and early identification of relapse.^18^

Among the age categories of children completing Ponseti treatment, the oldest age group had the shortest mean treatment duration, which could be explained by the fact that children enrolled at an older age required shorter brace duration, a fact supported by the literature.^8^

Regular follow-up over a period of several years is difficult, especially in low-resource settings where health systems are weak. Therefore, many studies are unable to report long-term outcomes conclusively,^1,5,21,36^ with reported loss to follow-up rates ranging between 0% and 32% in cohorts ranging from 17 to 307 patients.^15,28,37^ In our series of almost a thousand children, we initially encountered a very high loss to follow-up rate of more than 50%; however, with stringent programmatic guidelines of following up patients through phone calls and subsequent counseling, we were able to retrieve 40.2% of them. This highlights the importance of active involvement of the program team with parents, which influenced their decision to continue treatment. However, 32.0% of the enrolled children were still lost to follow-up, with most of them dropping out of the program in the first year of bracing. Therefore, increased focus on parental engagement in the initial few months of treatment may help improve adherence. Nearly half of the children lost to follow-up were not contactable, possibly because of change in mobile numbers. Therefore, even a simple strategy such as documenting two contact numbers instead of one may help mitigate this issue; this change was made in our program strategy toward the latter half of the study period. Relocation during the treatment period was cited as the reason for discontinuation of treatment, which could be addressed by a coordinated referral system, allowing children to receive treatment as close to home as possible. In this context, we have already established PQ clinics at other IHHN sites across Pakistan and hope that this will have a positive effect on treatment adherence.

Timely correction was achieved in more than four-fifths of the children completing Ponseti treatment. This success can be attributed to the rigorous program processes being followed efficiently by the program team, offering support and prompting early intervention and guidance to the parents in case of default. Conversely, interruption in treatment was identified as the major cause for delayed correction. Education of fathers was found to be markedly associated with the foot correction status, showing a positive relationship between the number of children achieving timely correction and the literacy level of the head of household. Literate parents have better understanding of the consequences of treatment failure, and their awareness leads to better compliance. In addition, male sex was also found to have a positive association with foot correction; however, this could simply be attributed to men enrolled in the program outnumbering women by threefold. It reflects the male sex prevalence of clubfoot globally, and we do not think that this represents a gender bias in seeking treatment.^1^

The Ponseti method is described to have a success rate of 90% to 95% in both short and long terms^6,24,37,38^; Morcuende et al^29^ reported a 98% success rate in clubfoot correction, whereas in our series, successful initial correction was achieved in 100% of the enrolled feet.

Strengths of this study include its large sample size, low relapse rate, strategies to recover patients from those lost to follow-up, absence of need for surgical correction, and excellent clinical outcomes in all treated children.

Threats to internal validity in this study include interobserver and intraobserver reliability because of the subjective nature of Pirani scoring.^39,40^ Because our program is donor-funded, it may not be generalizable or replicable in other resource-limited settings. In particular, good quality braces have been difficult to procure in many LMIC settings. Finally, attrition was notable in our study for reasons already described and needs to be addressed further.

Clubfoot treatment requires long-term follow-up to achieve good outcomes. A dedicated clubfoot program embedded within an orthopaedic service is effective in maintaining continuity of care and adherence to treatment.

## References

[1] Culverwell A Tapping C: Congenital talipes equinovarus in Papua New Guinea: A difficult yet potentially manageable situation. Int Orthop 2009;33:521-526. 18196240 10.1007/s00264-007-0511-x PMC2899095

[2] Dobbs MB Gurnett CA: Update on clubfoot: Etiology and treatment. Clin Orthop Relat Res 2009;467:1146. 19224303 10.1007/s11999-009-0734-9 PMC2664438

[3] Nogueira MP Fox M Miller K Morcuende J: The Ponseti method of treatment for clubfoot in Brazil: Barriers to bracing compliance. Iowa Orthop J 2013;33:161. 24027477 PMC3748873

[4] Sætersdal C Fevang JM Fosse L Engesæter LB: Good results with the Ponseti method: A multicenter study of 162 clubfeet followed for 2-5 years. Acta Orthop 2012;83:288-293. 22616746 10.3109/17453674.2012.693015 PMC3369157

[5] Spiegel DA Shrestha OP Sitoula P Rajbhandary T Bijukachhe B Banskota AK: Ponseti method for untreated idiopathic clubfeet in Nepalese patients from 1 to 6 years of age. Clin Orthop Relat Res 2009;467:1164-1170. 18987922 10.1007/s11999-008-0600-1 PMC2664412

[6] Staheli L: Clubfoot: Ponseti Management. Seattle, WA, Global HELP Organization, 2009.

[7] Morcuende JA: Congenital idiopathic clubfoot: Prevention of late deformity and disability by conservative treatment with the Ponseti technique. Pediatr Ann 2006;35:128132-130126. 10.3928/0090-4481-20060201-13 16493919

[8] Morcuende J Cook T. Clubfoot disability: Model for sustainable health systems programs in three countries. United States Agency for International Development (USAID) under Cooperative Agreement AID-OAA-A-11-00015; 2015.

[9] O'Shea RM Sabatini CS: What is new in idiopathic clubfoot? Curr Rev Musculoskelet Med 2016;9:470-477. 27696325 10.1007/s12178-016-9375-2 PMC5127955

[10] Owen RM Kembhavi G: A critical review of interventions for clubfoot in low and middle-income countries: Effectiveness and contextual influences. J Pediatr Orthop B 2012;21:59-67. 21811182 10.1097/BPB.0b013e3283499264

[11] Ponseti I Ponseti I: Common errors in the treatment of congenital clubfoot. Int Orthop 1997;21:137. 9195271 10.1007/s002640050137 PMC3616653

[12] Mkandawire NC Chipofya E Likoleche G Phiri M Katete L: Ponseti technique of correcting idiopathic clubfoot deformity. Malawi Med J 2003;15:99-101. 27528974 PMC3346032

[13] Shabtai L Specht SC Herzenberg JE: Worldwide spread of the Ponseti method for clubfoot. World J Orthop 2014;5:585. 25405086 10.5312/wjo.v5.i5.585 PMC4133465

[14] Owen RM Capper B Lavy C: Clubfoot treatment in 2015: A global perspective. BMJ Glob Health 2018;3:e000852. 10.1136/bmjgh-2018-000852 PMC6135438 30233830

[15] Lavy C Mannion S Mkandawire N : Club foot treatment in Malawi—A public health approach. Disabil Rehabil 2007;29:857-862. 17577720 10.1080/09638280701240169

[16] Tindall AJ Steinlechner CW Lavy CB Mannion S Mkandawire N: Results of manipulation of idiopathic clubfoot deformity in Malawi by orthopaedic clinical officers using the Ponseti method: A realistic alternative for the developing world? J Pediatr Orthop 2005;25:627-629. 16199944 10.1097/01.bpo.0000164876.97949.6b

[17] Mayne A Bidwai A Beirne P Garg N Bruce C: The effect of a dedicated Ponseti service on the outcome of idiopathic clubfoot treatment. Bone Joint J 2014;96:1424-1426. 25274932 10.1302/0301-620X.96B10.33612

[18] Harmer L Rhatigan J: Clubfoot care in low-income and middle-income countries: From clinical innovation to a public health program. World J Surg 2014;38:839-848. 24213946 10.1007/s00268-013-2318-9

[19] College of Physicians and Surgeons Pakistan: Orthopaedic Residency Training Programs. Karachi, Pakistan, CPSP, 2021. https://www.cpsp.edu.pk/fcps.php. Accessed August 11, 2021.

[20] Ayana B Klungsøyr PJ: Good results after Ponseti treatment for neglected congenital clubfoot in Ethiopia: A prospective study of 22 children (32 feet) from 2 to 10 years of age. Acta Orthop 2014;85:641-645. 25175659 10.3109/17453674.2014.957085 PMC4259042

[21] Smythe T Mudariki D Kuper H Lavy C Foster A: Assessment of success of the Ponseti method of clubfoot management in sub-Saharan Africa: A systematic review. BMC Musculoskelet Disord 2017;18:453. 29141609 10.1186/s12891-017-1814-8 PMC5688674

[22] Ponseti International Association. What is clubfoot? http://www.ponseti.info/what-is-clubfoot.html. Accessed 16 July, 2020.

[23] Sadruddin N Chinoy MA Javed MI: Soak the cast off. J Coll Physicians Surg Pak 2007;17:380-381. 17623597

[24] Evans AM Van Thanh D: A review of the Ponseti method and development of an infant clubfoot program in Vietnam. J Am Podiatr Med Assoc 2009;99:306-316. 19605924 10.7547/0980306

[25] Morcuende JA Abbasi D Dolan LA Ponseti IV: Results of an accelerated Ponseti protocol for clubfoot. J Pediatr Orthop 2005;25:623-626. 16199943 10.1097/01.bpo.0000162015.44865.5e

[26] Bensahel H Jehanno P Delaby J-P Themar-Noel C: Conservative treatment of clubfoot: The Functional method and its long-term follow-up. Acta Orthop Traumatol Turc 2006;40:181-186. 16757939

[27] Saltzman HM: Foot focus: International initiative to eradicate clubfeet using the Ponseti method. Foot Ankle Int 2009;30:468-471. 19439153 10.3113/FAI-2009-0468

[28] Lourenço A Morcuende J: Correction of neglected idiopathic club foot by the Ponseti method. J Bone Joint Surg Br 2007;89:378-381. 17356154 10.1302/0301-620X.89B3.18313

[29] Morcuende JA Dolan LA Dietz FR Ponseti IV: Radical reduction in the rate of extensive corrective surgery for clubfoot using the Ponseti method. Pediatrics 2004;113:376-380. 14754952 10.1542/peds.113.2.376

[30] Porecha MM Parmar DS Chavda HR: Mid-term results of Ponseti method for the treatment of congenital idiopathic clubfoot-(a study of 67 clubfeet with mean five year follow-up). J Orthop Surg Res 2011;6:3. 21226940 10.1186/1749-799X-6-3 PMC3031260

[31] Dobbs MB Gordon JE Walton T Schoenecker PL: Bleeding complications following percutaneous tendoachilles tenotomy in the treatment of clubfoot deformity. J Pediatr Orthop 2004;24:353-357. 15205614 10.1097/00004694-200407000-00002

[32] Burghardt RD Herzenberg JE Ranade A: Pseudoaneurysm after Ponseti percutaneous Achilles tenotomy: A case report. J Pediatr Orthop 2008;28:366-369. 18362805 10.1097/BPO.0b013e3181653b6f

[33] Ponseti IV: Relapsing clubfoot: Causes, prevention, and treatment. Iowa Orthop J 2002;22:55. 12180612 PMC1888384

[34] Hallaj-Moghaddam M Moradi A Ebrahimzadeh MH Habibzadeh Shojaie SR: Ponseti casting for severe club foot deformity: Are clinical outcomes promising? Adv Orthopedics 2015;2015:821690. 10.1155/2015/821690 PMC4338373 25755894

[35] Selmani E: Is Ponseti's method superior to Kite's for clubfoot treatment he? Eur Orthop Traumatol 2012;3:183-187.

[36] Mootha AK Saini R Krishnan V Kumar V Dhillon MS Bali K: Management of idiopathic clubfoot by the Ponseti technique: Our experience at a tertiary referral centre. J Orthop Sci 2011;16:184-189. 21298304 10.1007/s00776-011-0027-5

[37] Abdelgawad AA Lehman WB Van Bosse HJ Scher DM Sala DA: Treatment of idiopathic clubfoot using the Ponseti method: Minimum 2-year follow-up. J Pediatr Orthop B 2007;16:98-105. 17273035 10.1097/BPB.0b013e32801048bb

[38] Dyer P Davis N: The role of the Pirani scoring system in the management of club foot by the Ponseti method. J Bone Joint Surg Br 2006;88:1082-1084. 16877610 10.1302/0301-620X.88B8.17482

[39] Bettuzzi C Abati C Salvatori G Zanardi A Lampasi M: Interobserver reliability of Diméglio and Pirani score and their subcomponents in the evaluation of idiopathic clubfoot in a clinical setting: A need for improved scoring systems. J Childrens Orthop 2019;13:478-485. 10.1302/1863-2548.13.190010 PMC6808069 31695815

[40] Jillani S Aslam MZ Chinoy MA Khan MA Saleem A Ahmed SK: A comparison between orthopedic surgeon and allied health worker in pirani score. J Pak Med Assoc 2014;64(12 suppl 2):S127-S130. 25989760

